# Improving the performance of bioelectrochemical sulfate removal by applying flow mode

**DOI:** 10.1111/1751-7915.14157

**Published:** 2022-10-19

**Authors:** Shixiang Dai, Falk Harnisch, Mohammad Sufian Bin‐Hudari, Nina Sophie Keller, Carsten Vogt, Benjamin Korth

**Affiliations:** ^1^ Department of Environmental Microbiology Helmholtz Centre for Environmental Research GmbH – UFZ Leipzig Germany; ^2^ Department of Isotope Biogeochemistry Helmholtz Centre for Environmental Research GmbH – UFZ Leipzig Germany

## Abstract

Treatment of wastewater contaminated with high sulfate concentrations is an environmental imperative lacking a sustainable and environmental friendly technological solution. Microbial electrochemical technology (MET) represents a promising approach for sulfate reduction. In MET, a cathode is introduced as inexhaustible electron source for promoting sulfate reduction via direct or mediated electron transfer. So far, this is mainly studied in batch mode representing straightforward and easy‐to‐use systems, but their practical implementation seems unlikely, as treatment capacities are limited. Here, we investigated bioelectrochemical sulfate reduction in flow mode and achieved removal efficiencies (*E*_sulfate_, 89.2 ± 0.4%) being comparable to batch experiments, while sulfate removal rates (*R*_sulfate_, 3.1 ± 0.2 mmol L^−1^) and Coulombic efficiencies (*CE*, 85.2 ± 17.7%) were significantly increased. Different temperatures and hydraulic retention times (HRT) were applied and the best performance was achieved at HRT 3.5 days and 30°C. Microbial community analysis based on amplicon sequencing demonstrated that sulfate reduction was mainly performed by prokaryotes belonging to the genera *Desulfomicrobium*, *Desulfovibrio*, and *Desulfococcus*, indicating that hydrogenotrophic and heterotrophic sulfate reduction occurred by utilizing cathodically produced H_2_ or acetate produced by homoacetogens (*Acetobacterium*). The advantage of flow operation for bioelectrochemical sulfate reduction is likely based on higher absolute biomass, stable pH, and selection of sulfate reducers with a higher sulfide tolerance, and improved ratio between sulfate‐reducing prokaryotes and homoacetogens.

## Graphical abstract

In this study, continuous mode was applied to initial autotrophic bioelectrochemical sulfate reduction allowing a tripling of the sulfate removal rate compared to batch mode conditions which had been applied in previous studies. Microbial community analysis demonstrated the presence of a few hydrogenotrophic and heterotrophic sulfate reducers but also an increased abundance of homoacetogens, a process which is seemingly beneficial for bioelectrochemical sulfate reduction as the produced acetate could lead to an improved biomass production.

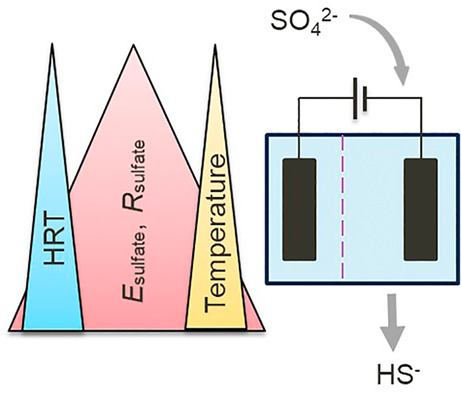

## INTRODUCTION

Many industrial processes produce sulfate‐rich wastewater, and its excessive discharge can affect environmental and human health, thus calling for proper treatment (Hao et al., 2014). A sustainable alternative to conventional physiochemical treatment technologies (e.g., reverse osmosis (Biesheuvel et al., 2019), ettringite formation (Pratinthong et al., 2021), or barium precipitation (Xu et al., 2021)) is biological sulfate removal. Under anoxic conditions, sulfate‐reducing prokaryotes (SRP) use sulfate as terminal electron acceptor, that is, dissimilatory sulfate reduction, leading to the formation of bisulfide (HS^−^) and volatile hydrogen sulfide (H_2_S, Equation 1) at pH‐neutral conditions (Muyzer & Stams, 2008). Both chemical species are most often summarized under the terminus sulfide as also done within this article. Subsequently, the generated sulfide can be oxidized to other valuable forms like elemental sulfur.

(1) $${\mathrm{SO}}_{4}^{2-}+8e^{-}+9H^{+}\to HS^{-}+4H_{2}O$$

As sulfate‐rich wastewaters from pulp and paper plants, mining, and the pharmaceutical industry are typically deficient in organics (Blazquez et al., 2016), additional electron donors like H_2_ (for autotrophic SRP), volatile fatty acids (Hao et al., 2014) or carbohydrates (Zhao et al., 2020) (both for heterotrophic SRP) need to be supplemented to drive the reduction of sulfate. This considerably increases operational expenditures (opex; Liamleam & Annachhatre, 2007). Notably, many hydrogenotrophic SRP need acetate in addition to CO_2_ for growth (Rabus et al., 2015).

Primary microbial electrochemical technologies (MET) are based on electroactive microorganisms (EAM; Logan et al., 2019) that use electrodes as inexhaustible electron sources and electron sinks. MET have been proposed for treating wastewater (Min & Logan, 2004), groundwater (Pous et al., 2018), and surface water (Ramírez‐Vargas et al., 2018) from numerous contaminants like aromatic compounds, sulfate, and metal ions (i.e., microbial electroremediation; Wang et al., 2020). The electrodes are introduced in bioelectrochemical systems (BES) facilitating a controllable electron transfer between EAM and electrodes, which can either occur directly at the electrode surface, e.g., via membrane‐bound cytochromes (Lovley, 2011) or indirectly via redox mediators like riboflavin and H_2_ (Kumar et al., 2017). In microbial electrolysis cells (MEC; Zhang & Angelidaki, 2014) commonly used for microbial electroremediation, the electrode potential is adjusted for steering electron transfer reactions. For treating sulfate‐rich wastewater lacking electron donors, MEC represent a promising opportunity to achieve sulfate removal (Agostino & Rosenbaum, 2018) using biotically (Rozendal et al., 2008) or abiotically (Xiu et al., 2019) produced H_2_ from the cathode as electron donor for autotrophic SRP.

Autotrophic sulfate reduction facilitated by electrochemical hydrogen production was previously investigated with different experimental setups achieving sulfate reduction rates of up to 8.2 ± 1.1 mmol L^−1^ day^−1^ (Blazquez et al., 2017; Luo et al., 2020; Pozo et al., 2015) by applying a cathode potential of −1.1 V (vs. standard hydrogen electrode [SHE]). Furthermore, Dai et al. (2022) studied the sulfate reduction in one‐chamber and two‐chamber BES operated in batch‐mode identifying the latter as advantageous as anodic re‐oxidation of sulfide is avoided. In Dai et al. (2022) we used an identical experimental setup as in this study for achieving a sulfate reduction rate and an electron recovery efficiency of 1.0 ± 0.3 mmol L^−1^ day^−1^ and 83.9 ± 1.3%, respectively. In the majority of studies, batch and fed‐batch systems were applied which have a rather limited application potential for treating sulfate‐contaminated waters. In contrast, a MEC in flow mode is more relevant for field applications due to its higher absolute removal rates.

To our knowledge, only Coma and colleagues (Coma et al., 2013) studied bioelectrochemical sulfate reduction in flow mode with a cathodic hydraulic retention time (HRT) of 0.28 days and internal recirculation. They reported a sulfate removal rate of 0.024 mmol L^−1^ day^−1^ which seems low compared to the rates reported in batch and fed‐batch studies (Blazquez et al., 2016, 2017; Luo et al., 2020), ranging from 0.4 to 8.2 mmol L^−1^ day^−1^.

To close this knowledge gap, we systematically investigated MEC in flow mode in terms of bioelectrochemical sulfate reduction, the influence of the operational parameters HRT and temperature, and the structures of the process‐performing microbial community at different operational stages.

## EXPERIMENTAL PROCEDURES

### Bioelectrochemical systems design and operation

Experiments were performed as duplicates in four‐neck round‐bottom flasks with a total/working volume of 350/250 ml in a two‐chamber configuration (cation exchange membrane: fumasep®FKE, FuMA‐Tech GmbH, Germany) as described previously (Dai et al., 2022; Figure S1). Both anode and cathode were Pt‐covered titanium electrodes (PLATINODE®, Umicore Electroplating, Schwaebisch Gmuend, Germany; cathode: 10 cm^2^; anode: 6 cm^2^) spot‐welded to a titanium wire. An Ag/AgCl reference electrode (Ag/AgCl sat. KCl, +0.197 V vs. SHE, SE11, Xylem Analytics Germany Sales GmbH & Co. KG Sensortechnik Meinsberg, Germany) was used. All provided potentials refer to the SHE by conversion from Ag/AgCl sat. KCl reference electrodes. Two needles were pierced through chloroprene stoppers (Deutsch & Neumann GmbH, Hennigsdorf, Germany) as influent and effluent ports, connected to medium/waste bottles. Anoxic mineral salt medium (MSM) buffered with CO_2_/NaHCO_3_ (30 mM) was used for all experiments (Dai et al., 2022). In brief, the basal MSM was flushed with N_2_ to remove oxygen. Each liter basal MSM was anaerobically supplemented with 30 ml of a CO_2_‐saturated 1 M NaHCO_3_ solution and 3 ml trace element solution within an anaerobic chamber. The sulfate‐reducing enrichment culture used in this study was obtained from sediment of a freshwater pond (51°20′12.2″ N 12°25′51.0″ E). 10 g of sediment, 50 ml MSM, 10 mM DL‐lactate, 3 sterile Fe(0) nails, and 10 mM sulfate were anaerobically incubated at 30°C in 100 ml serum bottles closed with butyl stoppers. The culture was transferred biweekly. For inoculation of the BES, the whole culture volume was centrifuged (10,000 *g*, 10 min) and re‐suspended in 15 ml anoxic MSM. The counter electrode chamber was filled with 40 ml anoxic MSM without sulfate.

Both BES were stirred (400 rpm). Cathodes were poised at −0.8 V (multipotentiostat MPG‐2, Bio‐Logic Science Instruments, France) mainly promoting the hydrogen evolution reaction, and thus allowing hydrogenotrophic sulfate reduction (Dai et al., 2022). Sulfate reduction during continuous operation (sulfate concentration in the influent, *C*_SO__4__2__−__,__in_  = 13.9 ± 1.3 mM) was investigated by changing HRT and temperature (Table 1).

**TABLE 1:** Overview of conducted experiments, varied process conditions (HRT, hydraulic retention time), and main process parameters (*E*_sulfate_, sulfate removal efficiency; *R*_sulfate_, sulfate removal rate; *CE*, Coulombic efficiency).

No.	Experimental phase	Duration (days)	HRT (days)	Temperature (°C)	*E*_sulfate_ (%)	*R*_sulfate_ (mM L^−1^ day^−1^)	*CE* (%)
1	Inoculation	0–11	—	30	—	—	—
2	Adaptation	11–107	2.5	30	47.4 ± 7.7	2.3 ± 0.3	27.8 ± 5.8
3	Influence of HRT	107–137	1.5	30	20.8 ± 0.2	2.0 ± 0.1	45.6 ± 3.2
4		137–183	2.5	30	40.8 ± 3.8	2.1 ± 0.1	27.0 ± 0.9
5		183–223	3.5	30	89.2 ± 0.4	3.1 ± 0.2	85.2 ± 17.7
6	Influence of temperature	223–233	3.5	20	62.3 ± 3.1	1.8 ± 0.2	66.7 ± 26.4
7		233–243	3.5	14	56.1 ± 0.9	1.8 ± 0.3	58.4 ± 9.4
8		243–253	3.5	20	57.9 ± 1.3	1.8 ± 0.3	61.4 ± 6.2
9	253–261	3.5	30	83.3 ± 2.6	3.0 ± 0.6	94.1 ± 24.9

### Experimental design, BES sampling, and data analysis

Sulfate reduction during continuous operation was investigated by changing HRT and temperature (Table 1). During the whole study, BES were regularly sampled (every 2–4 days) to measure sulfate, sulfide, *OD*_600_, and pH (Appendix S1). At the end of every experimental phase, planktonic cells were harvested by collecting effluents. Biofilm samples were collected from the cathode at the end of this study and were analysed via amplicon sequencing of bacterial 16S rRNA gene (Appendix S2). *R*_sulfate_, *E*_sulfate_, and sulfide formation were calculated considering sulfate and sulfide concentrations in influent and effluent, flow rate, and BES working volume (Appendix S3).

## RESULTS AND DISCUSSION

### Influence of HRT and temperature on sulfate removal

After inoculation, BES were operated for more than 100 days to obtain steady‐state conditions (<5% deviation of *E*_sulfate_ for three consecutive measurements). Subsequently, the influence of HRT and temperature on bioelectrochemical sulfate reduction was studied. The comparable long period to achieve a steady state points out one experimental limitation throughout the whole study: as the medium contained trace amounts of iron, sulfate reduction to sulfide resulted in FeS precipitates. Thus, the tubing was regularly changed to prevent clogging. This is an important lesson learned for the experimental design of future studies. Several measures are conceivable to overcome this pitfall, for instance, separation of microbial growth phase and sulfate reduction phase (i.e., utilizing different iron requirements), improved iron/sulfide ratio (by applying sulfate dosing and an optimized flow rate; Nielsen et al., 2008), keeping pH at slightly acidic conditions, and more appropriate tubing. Transferring this into practice, one needs to consider FeS precipitation as it could lead to increased opex due to decreased performance and increased maintenance requirements. This accounts most prominently for 3D electrodes (e.g., bed cathodes; Kerzenmacher, 2019), which were proposed as efficient tool for microbial electroremediation. Furthermore, the corrosion potential of sulfide needs to be considered (Little et al., 2020).

**FIGURE 1:**
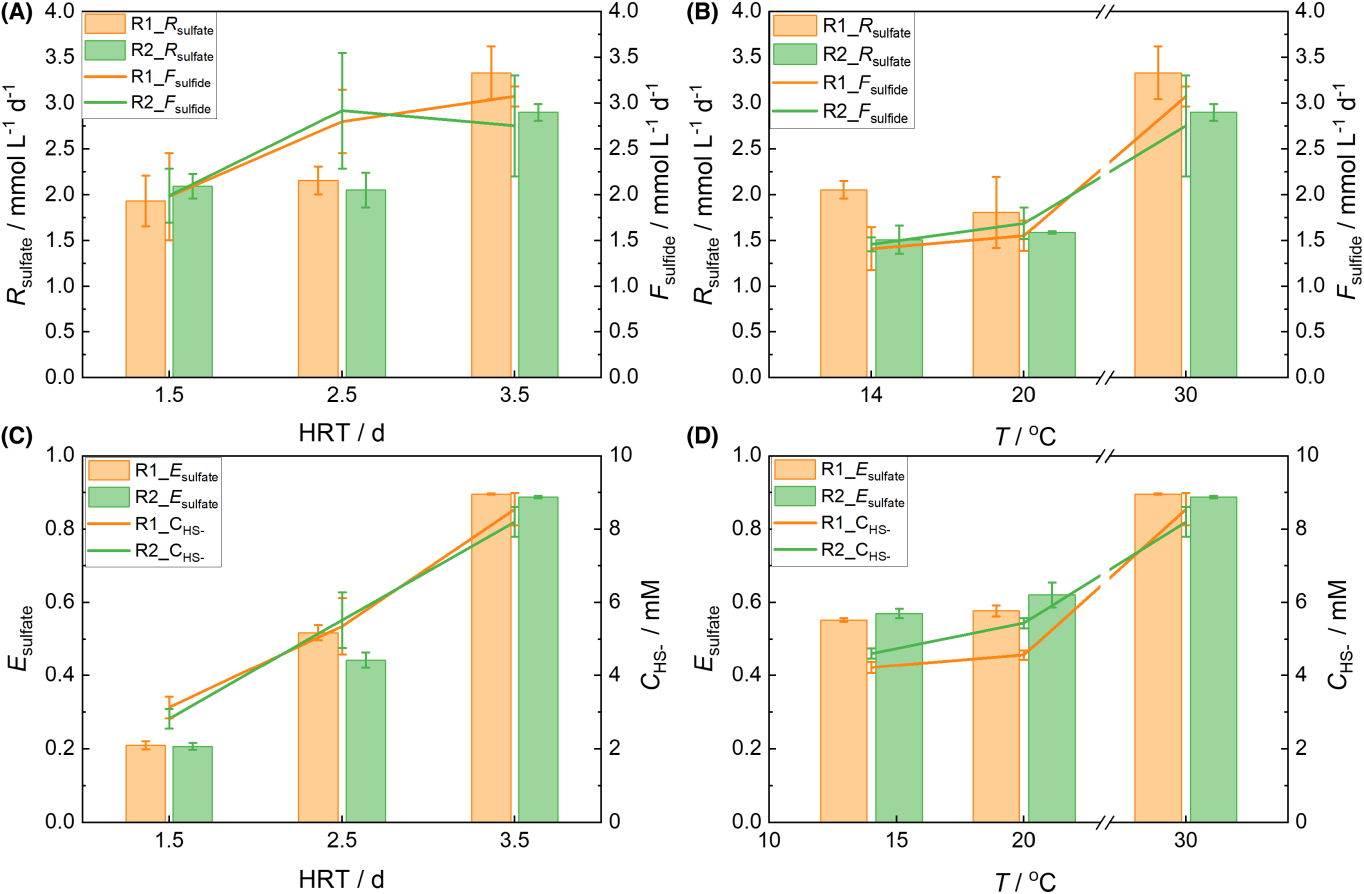
Performance of bioelectrochemical systems (BES) at different hydraulic retention times (HRT) and temperatures (*T*). Sulfate removal rates (*R*_sulfate_) and sulfide formation rates (*F*_sulfide_) at different HRT at 30°C (A) and at different temperatures at HRT of 3.5 days (B). Sulfate removal efficiencies (*E*_sulfate_) and sulfide concentrations (*C*_HS−_) at different HRT at 30°C (C) and at different temperatures at HRT of 3.5 days (D). The error bars represent standard deviations (SD) calculated from three consecutive measurements representing steady‐state conditions (SD of sulfate removal efficiency <5%).

The highest sulfate removal rate (*R*_sulfate_) and removal efficiency (*E*_sulfate_) with 3.1 ± 0.2 mmol L^−1^ day^−1^ and 89.2 ± 0.4%, respectively, were achieved at HRT 3.5 days and 30°C (Figure 1A,C; Table 1). Lowering the HRT to 2.5 days resulted in decreased *R*_sulfate_ and *E*_sulfate_ of 2.1 ± 0.1 mmol L^−1^ day^−1^ and 40.8 ± 3.8%, respectively. The obtained *R*_sulfate_ were substantially higher compared to the previously performed batch experiments with the same BES (1.0 ± 0.3 mmol L^−1^ day^−1^) indicating a general advantage of flow operation for sulfate reduction compared to batch operation (see discussion below). As the abiotic controls of the batch experiments did not show any sulfate reduction, corresponding controls were omitted in the present study (Dai et al., 2022). Considering that the dilution rates (0.012, 0.017, and 0.028 h^−1^ at HRT of 3.5, 2.5, and 1.5 days, respectively) were generally lower than many reported maximum growth rates of hydrogenotrophic and heterotrophic SRP (0.029–0.41 h^−1^; Badziong & Thauer, 1978; Elferink et al., 1998; Oude Elferink, 1998), it was challenging to identify SRP activity as bottleneck at lower HRT. Similarly, the growth rate of homoacetogens (e.g., 0.14 h^−1^; Morinaga & Kawada, 1990) presumably supporting bioelectrochemical sulfate reduction in this study (see below and Dai et al. (2022)) seemingly did not limit the overall performance. Further decreasing HRT to 1.5 days did not affect *R*_sulfate_ (2.0 ± 0.1 mmol L^−1^ day^−1^) compared to 2.5 days HRT but resulted in a lower *E*_sulfate_ of 20.8 ± 0.2% (Figure 1A,C). The comparable current densities (i.e., electron donor supply rate, Figure S2) for HRT 3.5 and 1.5 days indicate that downstream processes like, for example, H_2_ solubility and mass transfer, H_2_ uptake rate, or microbial metabolism limited the BES performance. Nevertheless, the low *E*_sulfate_ during HRT of 1.5 days was still in the range of previously reported results, for instance, *E*_sulfate_ 18.0 ± 8.8% (HRT 1 day, 30°C; Sangcharoen et al., 2015) and less than 20% (HRT 10 days; Zhang et al., 2020). The observed Coulombic efficiencies (*CE*) at different HRT support the suggested bottlenecks of continuous operation of bioelectrochemical sulfate removal. The *CE* of 85.2 ± 17.7% at HRT 3.5 days (Table 1) is in the same range compared to the previous batch experiment (83.9 ± 1.3%) and thus considerably higher than usually reported literature values which are around 50% (Dai et al., 2022). *CE* decreased at HRT 2.5 days and 1.5 days achieving 27.0 ± 0.9% and 45.6 ± 3.2%, respectively, indicating the washout of H_2_ before it could be consumed by the microorganisms and thus the loss of electron donor. We speculate that the more pronounced *CE* decrease at HRT 2.5 days compared to 1.5 days was due to an interim oxygen intrusion scavenging electrons from the cathode. These results suggest that in case of upscaling of bioelectrochemical sulfate reduction, design parameters like electrode area‐to‐reactor volume ratio and BES stacking are more promising for an efficient process than faster flow rates (i.e., higher sulfate loads) which was, for instance, successfully demonstrated for bioelectrochemical nitrate removal (Pous et al., 2017).

For testing more realistic field application scenarios (i.e., groundwater treatment), the temperature was gradually lowered while keeping HRT at 3.5 days so that a certain range of the global groundwater temperatures was covered within this study (Benz et al., 2017). *R*_sulfate_ decreased by 45% to 1.8 ± 0.2 mmol L^−1^ day^−1^ at 20°C, roughly following the Arrhenius equation, which estimates a decrease by a factor of 2–3 for 10°C decrease in temperature. However, the *R*_sulfate_ in case of 14°C (1.8 ± 0.3 mmol L^−1^ day^−1^) was comparable to 20°C (Figure 1B, Table 1), indicating that mass transfer processes rather than chemical, electrochemical, or biological reactions were limiting the overall process. Similarly, *E*_sulfate_ decreased to 62.3 ± 3.1% at 20°C but no further significant decrease was observed for 14°C (56.1 ± 0.9%). As the current density remained nearly constant within the tested temperature range (Figure S2), it seems likely that the observed performance decrease at 20°C was caused by a decreased biological activity. However, the similar performances of *R*_sulfate_ and *E*_sulfate_ at 20°C and 14°C suggest either an activity plateau of the microbiota in this temperature range or a mass transfer‐limiting process (e.g., H_2_ solubility). *CE* corresponded to the observed sulfate removal parameters with the highest *CE* at 30°C (85.2 ± 17.7% and 94.1 ± 24.9%, Table 1) and considerably lower values at 20°C (66.7 ± 26.4% and 61.4 ± 6.2%) and 14°C (58.4 ± 9.4%) suggesting that lower biological activities at a lower temperature resulted in a washout of hydrogen before it could be consumed.

**FIGURE 2:**
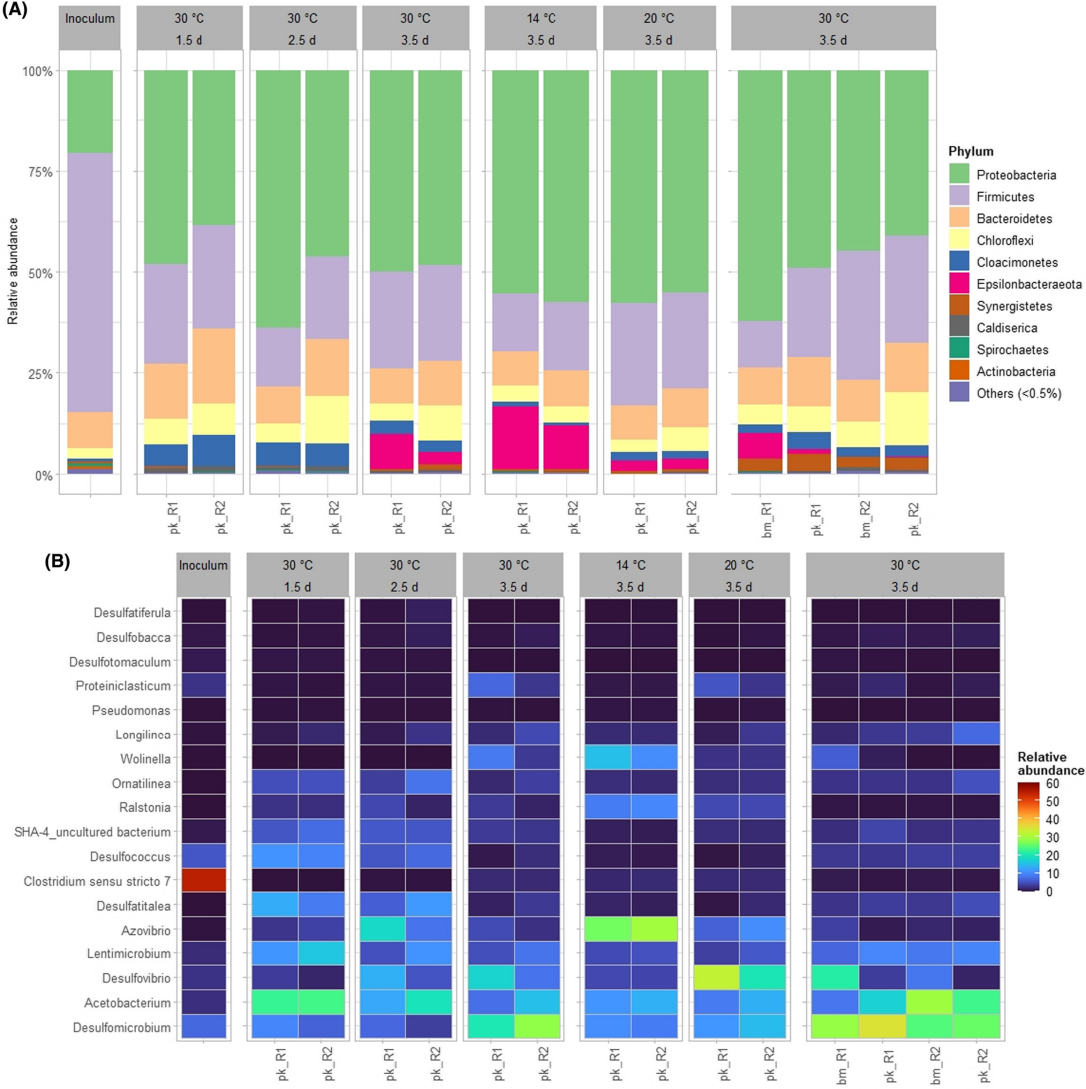
Taxonomic classification of the dominant phylogenetic groups of bioelectrochemical systems (BES, reactor 1 [R1] and reactor 2 [R2]) performing sulfate reduction at phylum level (A) and genus level (B). pk_R1 and pk_R2 represent samples derived from the liquid phase of BES. bm_R1 and bm_R2 represent biofilm samples that were taken at the end of experiment.

The reproducibility of BES performances at same process conditions being apart in time proved the robustness of bioelectrochemical sulfate removal against different process conditions. For instance, HRT 2.5 days and 30°C were applied twice with one month's operation at HRT 1.5 days in between, and experimental phases were comparable (e.g., *R*_sulfate_ were 2.3 ± 0.3 and 2.1 ± 0.1 mM L^−1^ day^−1^ during phases 2 and 4, respectively, Table 1). The good reproducibility is even more pronounced in case of HRT 3.5 days and 30°C as all process parameters of phase 9 achieved similar values compared to phase 5 within 1 week (Table 1), although the microbial composition was significantly different in the phases between (Figure 2).

Across all experimental conditions, *R*_sulfate_ and sulfide formation rates (*F*_sulfide_) corresponded, resulting in a sulfur balance of 94.6 ± 12.5% (*R*_sulfate_ / *F*_sulfide_) averaging all experimental conditions. This indicates only minor sulfur losses via FeS precipitation and H_2_S volatilization. These minor sulfur losses were surprising, considering the volatility of hydrogen sulfide at neutral conditions (Saad et al., 2021) and the considerable formation of FeS during the experiment. This finding is promising as the formed sulfide can be partially oxidized and recovered to elemental sulfur by subsequent chemical or biological methods (Cai et al., 2017; Rabbani et al., 2015; Saad et al., 2021). *F*_sulfide_ was considerably higher than *R*_sulfate_ only at HRT 2.5 days (73.6% *R*_sulfate_ / *F*_sulfide_) even though the rather huge error reduces its significance. We speculate that this was caused by analytical variability and to a lesser extent to pH‐dependent FeS precipitation and H_2_S volatilization reactions. The slight pH shift from 7.5 to 7.2 when the HRT was changed from 3.5 to 2.5 days (Figure S4A) may have additionally produced a small release of sulfide due to FeS dissolution (Rickard, 2006) leading to higher apparent *F*_sulfide_.

The highest sulfide concentration (*C*_HS−_ = 8.4 mM) was reached at 30°C and HRT 3.5 days. In accordance to *R*_sulfate_, *C*_HS−_ decreased to 5.4 and 3.0 mM at HRT 2.5 and 1.5 days, respectively. *C*_HS−_ amounted to 5.0 and 4.4 mM at 20°C and 14°C, respectively. Literature reports 50% lower SRP activity at *C*_HS−_ of 7–9 mM (Koschorreck, 2008). Therefore, it is conceivable that the achieved *C*_HS−_ influenced *R*_sulfate_ for all experimental conditions demanding substantial conceptual and engineering efforts (e.g., HRT adaptation, sulfate dosing) for a successful transfer into practice. However, it is of note that the highest *C*_HS−_ within this study (8.4 mM at HRT 3.5 days and 30°C) was accompanied by a substantially higher *R*_sulfate_ compared to previous batch experiments with a lower *C*_HS−_ (6.8 ± 1.1 mM, Dai et al., 2022) indicating the selection of hydrogen‐oxidizing freshwater sulfate reducers exhibiting higher sulfide tolerance as it was already demonstrated for sulfate‐reducing communities (Icgen & Harrison, 2006).

One promising aspect is the comparable low mass‐related power consumption of this proof‐of‐concept study, which amounted to 0.66 kWh kg_sulfate_^−1^ at HRT 3.5 days and 30°C considering only the spent power for sulfate reduction. This is the lowest value of tested conditions (Figure S3) and is in the same order of magnitude as established treatment technologies like gypsum precipitation (0.23–26 kWh kg_sulfate_^−1^) and ettringite precipitation (0.31–36 kWh kg_sulfate_^−1^) which additionally require input of chemicals (Kinnunen et al., 2017). Moreover, the power consumption was orders of magnitude lower than in previous batch experiments (14.9 kWh kg_sulfate_^−1^) and electrokinetic approaches (248.9 kWh kg_sulfate_^−1^; Annamalai et al., 2015; Appendix S4).

### Evolution of the microbial community during different experimental conditions

The microbial community of all samples across all experimental conditions (including inoculum) were dominated by the phyla Proteobacteria, Firmicutes, and Bacteroidetes representing in sum 86.4 ± 5.0%. Notably, Proteobacteria were enriched in the BES experiments reaching an abundance of 52.2 ± 8.3% compared to 20.7% in the inoculum. Concomitantly, the abundance of Firmicutes decreased from 64.1% in the inoculum to 22.6 ± 5.1% in the BES (Figure 2A).

The inoculum consisted of different SRP from the genera *Desulfomicrobium*, *Desulfovibrio*, *Desulfococcus*, *Desulfotomaculum*, *Desulfobacca*, and *Desulfatiferula* with a total abundance of 16.4% (Rabus et al., 2015). During cultivation in BES, *Desulfomicrobium* and *Desulfovibrio* were considerably enriched, reaching an abundance of 17.0 ± 9.7% and 10.5 ± 9.0%, respectively, across all samples and cultivation conditions (Figure 2B). Both genera were already identified in several hydrogenotrophic sulfate‐reducing systems (Dias et al., 2008). In addition, the presence of *Desulfovibrio* in BES was proven few times (Blazquez et al., 2019; Hu et al., 2018; Luo et al., 2020). At 14°C (9.9%) and 20°C (13.3%), the abundance of *Desulfomicrobium* decreased compared to 30°C (24.5%). At 20°C, this was compensated by *Desulfovibrio* showing an abundance increase from 30°C (13.0 ± 4.7%) to 20°C (26.5 ± 6.3%). Therefore, the total SRP abundance was similar at 20°C (41.7 ± 1.5%) and 30°C (41.7 ± 3.3%) suggesting functional redundancy (Koch et al., 2018) and the microbiota's capability to adapt to changing HRT and temperature. Nevertheless, the observed decline in *R*_sulfate_ when changing temperature from 30°C to 20°C either suggests a decreased sulfate reduction capability due to the composition change of the microbiota or a general decline of SRP due to washout, as it can be anticipated from the *OD*_600_ decrease when comparing measurements at 20°C and 30°C (Figure S4B). Although the SRP abundance drastically decreased to 16.5 ± 1.2% when the temperature was changed to 14°C, *R*_sulfate_ remained rather constant. We speculate about a change in reaction mechanisms (please see below for discussion about microaerobes). In total, SRP accumulated to 36.5 ± 10.7% averaging all experimental conditions. This contrasted with our previous study on bioelectrochemical sulfate reduction in batch operation that observed a SRP abundance of 73.8 ± 0.2% in the biofilm phase (Dai et al., 2022). Obviously, SRP were constantly washed out during flow operation indicating that growth conditions were not optimal as reported maximum growth rates of SRP are substantially higher than applied dilution rates (Elferink et al., 1998). However, it is interesting to see that the flow mode achieved a substantially higher *R*_sulfate_ of 3.1 ± 0.2 mmol L^−1^ day^−1^ (3.5 days, 30°C) with a SRP abundance of 41.6 ± 3.4% compared to batch operation (*R*_sulfate_ = 1.0 ± 0.3 mmol L^−1^ day^−1^) with higher SRP abundance (49.9 ± 11.7%; Dai et al., 2022). This indicates that continuous operation is advantageous for bioelectrochemical sulfate reduction compared to batch operation. However, it should be considered that comparisons of batch and flow operation might be biased by dead or inactive cells in batch experiments which are washed out in case of flow conditions. Furthermore, it needs to be stressed that the interpretation of structure–function relationships of the microbial community were solely derived from 16S rRNA amplicon sequencing not providing information on the metabolic activity of the microorganisms. Nevertheless, the enrichment of SRP during the different experimental conditions compared to the inoculum indicates that SRP were actively growing performing sulfate reduction. Seemingly, the stable neutral pH during continuous operation (7.5 ± 0.3, Figure S4) was beneficial for hydrogen uptake of SRP bioelectrochemical sulfate (Blazquez et al., 2017; Fauque et al., 1987). However, in a real‐world application scenario, the sulfate‐containing wastewater or groundwater likely possess neither the optimal pH nor a high buffer capacity (Hao et al., 2014) so that occurring pH changes probably influence SRP activity.

Interestingly, the abundances of *Azovibrio* (28.3%), *Ralstonia* (9.6%), and *Wolinella* (13.0%) increased at 14°C (Figure 2B; Figure S5) indicating changes in process reactions. *Azovibrio* and *Ralstonia* are typical microaerobes (Reinhold‐Hurek & Hurek, 2000; Volova & Voinov, 2003), which can likely use acetate and H_2_ as electron donors (Cramm, 2009). Therefore, microaerobic reactions seemingly played an increased role at 14°C. *Wolinella* can reduce polysulfides using H_2_ as electron donor (Jankielewicz et al., 1994), which could lead to sulfur cycle in BES (Ringel et al., 1996). *Azovibrio*, *Ralstonia*, and *Wolinella* were also present in lower abundance at other temperatures; hence some of the hydrogen and acetate always seemed to be channelled (Cramm, 2009; Volova & Voinov, 2003) to other electron acceptors than sulfate (e.g., oxygen, polysulfides).

The observed higher *R*_sulfate_ compared to the batch experiments could also result from improved cross‐feeding within the microbial community. The abundance of the homoacetogenic genus *Acetobacterium* considerably increased to 17.2 ± 6.3% for all conditions (Figure 2B) compared to batch mode (0.9 ± 0.3%; Dai et al., 2022), indicating substantial acetate production from CO_2_ and cathodically produced H_2_, which supported growth of SRP and consequently sulfate reduction in BES (Omar et al., 2018). Although interpretation of *OD*_600_ data was challenging as FeS particles influence the measurements, comparing *OD*_600_ values between batch (0.05 ± 0.03) and flow (0.22 ± 0.07) mode (Figure S4) indicated higher absolute biomass in the present study, possibly balancing the lower relative abundances of SRP. A further indicator for a complex food web within the BES performing sulfate reduction is the enrichment of the genus *Lentimicrobium*, reaching an abundance of 8.2 ± 3.3% in all BES samples. *Lentimicrobium* is reported as strictly anaerobic chemoorganotrophic eubacterium that cannot grow with acetate only but needs yeast extract and other carbon sources like pyruvate, suggesting either growth by using dead cell materials or the exchange of further metabolites beyond acetate within the cultivated microbiota (Sun et al., 2016).

## CONCLUSION

To summarize, we showed that bioelectrochemical sulfate reduction in flow mode can achieve higher sulfate reduction rates compared to batch experiments in an identical BES. Thereby, the highest *R*_sulfate_ and *E*_sulfate_ of 3.1 ± 0.2 mmol L^−1^ day^−1^ and 89.2 ± 0.4%, respectively, were achieved at HRT 3.5 days and 30°C while CE reached 85.2 ± 17.7%. Interestingly, this superior sulfate reduction rate was accompanied by a 50% decrease of SRP abundance, indicating the advantages of flow operation possibly due to selection of sulfate reducers with higher sulfide tolerance, stable pH, and higher absolute biomass. Furthermore, continuous operation led to an increased abundance of homoacetogens, which support bioelectrochemical sulfate reduction via acetate production, as many SRP require organics for growth. Consequently, the reduction in process efficiency by hydrogen consumption of homoacetogens is compensated by an increased process stability. This suggests that a complex food web was developed that improved bioelectrochemical sulfate reduction in the researched BES. The promising results open a very clear perspective towards application, as more relevant reduction rates seem conceivable.

## AUTHOR CONTRIBUTIONS

*Conception/design of the study*: Shixiang Dai, Benjamin Korth, Carsten Vogt and Falk Harnisch. *Experimental data collection*: Shixiang Dai. *Amplicon sequencing*: Mohammad Sufian Bin‐Hudari, Nina Sophie Keller and Shixiang Dai. *Data analysis and interpretation*: Shixiang Dai, Nina Sophie Keller, Mohammad Sufian Bin‐Hudari and Benjamin Korth. *Manuscript draft*: Shixiang Dai. *Final revision of the manuscript*: Benjamin Korth, Carsten Vogt and Falk Harnisch.

## FUNDING INFORMATION

China Scholarship Council (Grant / Award Number: CSC201804910500).

## CONFLICT OF INTEREST

The authors declare that they have no known competing financial interests or personal relationships that could have appeared to influence the work reported in this paper.

## Supporting information


Appendix S1–S4
Figures S1–S5
