# Supplementary material for: Improving the performance of bioelectrochemical sulfate removal by applying flow mode
Source: Microb Biotechnol. 2022 Oct 19;16(3):595–604. doi: 10.1111/1751-7915.14157 (PMC9948226; doi:10.1111/1751-7915.14157)
Supplement: Supplementary file 1 — Appendix S1–S4 Figures S1–S5 [file MBT2-16-595-s001.docx]

**Supplementary Material**

Improving the performance of bioelectrochemical sulfate removal by applying flow mode

Shixiang Dai^a^, Falk Harnisch^a^, Mohammad Sufian Bin-Hudari^b^, Nina Sophie Keller^b^, Carsten Vogt^b^, and Benjamin Korth^a,^*

^a^Department of Environmental Microbiology, Helmholtz Centre for Environmental Research GmbH - UFZ, Permoserstr. 15, 04318 Leipzig, Germany

^b^Department of Isotope Biogeochemistry, Helmholtz Centre for Environmental Research GmbH - UFZ, Permoserstr. 15, 04318 Leipzig, Germany

[shixiang.dai@ufz.de](mailto:shixiang.dai@ufz.de), [falk.harnisch@ufz.de](mailto:falk.harnisch@ufz.de), [mohammad-sufian.bin-hudari@ufz.de](mailto:mohammad-sufian.bin-hudari@ufz.de), [nina-sophie.keller@ufz.de](mailto:nina-sophie.keller@ufz.de), [carsten.vogt@ufz.de](mailto:carsten.vogt@ufz.de)

*Correspondence:

[benjamin.korth@ufz.de](mailto:benjamin.korth@ufz.de), +49 341 235-1378, ORCID iD: 0000-0002-5105-2439

**SI-1 Chemical analyses**

Every 2-3 days, 5 mL reactor liquid was anaerobically collected using integrated sampling ports.

For sulfate measurement, 4 mL of liquid was transferred into test tubes (Eppendorf SE, Germany) and directly deep-frozen with liquid nitrogen and stored at −80 °C until analysis. From the remaining 1 mL liquid, 0.2 mL were immediately transferred into 1 mL zinc acetate (3 wt %) for bisulfide measurement via the modified methylene blue method described elsewhere (Bin Hudari, et al., 2020). The remaining 0.8 mL liquid was used for *OD*_600_ and pH measurement. The pH was measured using a pH meter (SevenCompact S220, Mettler Toledo GmbH, Germany) and *OD*_600_ using a spectrophotometer (U-2000, Hitachi High-Tech Corporation, Japan).

Sulfate was quantified by ion chromatography (IC) using a Dionex ICS-6000 ion chromatograph equipped with a conductivity detector (suppressed conductivity, ADRS 600, 2 mm) and an IonPac AG18 column (2 mm, ThermoScientific, USA). Potassium hydroxide was used as eluent with a flow rate of 0.25 mL min^−1^ for 14 min at 30 °C.

**SI-2 Microbial community analysis**

At the end of every experimental condition, the liquid phase was sampled and planktonic cells were analyzed by amplicon sequencing allowing to investigate the impact of HRT and temperature on the microbial composition. 8 mL reactor liquid were taken and centrifuged (10 min, 4 °C, and 10,000×g). Subsequently, the cell pellets were collected. In addition, the cathode surface was sampled at the end of the experiment to investigate the composition of the cathodic biofilm. Pellets of centrifuged liquid samples and biofilm samples were stored at −30 °C until further analysis. Genomic DNA from biofilms and planktonic cells was extracted using the NucleoSpin® kit for soil (Macherey-Nagel GmbH & Co. KG, Germany) according to the manufacturer’s instruction. Fluorometric quantification of extracted DNA was performed using an Invitrogen™ Qubit™ Fluorometer and Qubit™ dsDNA HS Assay (Thermo Fisher Scientific Inc., U.S.A.) according to manufacturer’s instructions.

For sequencing, 16S rRNA gene was amplified using the Klindworth primer-set (S-D-Bact-341-b-S-17/S-D-Bact-0785-a-A-21) targeting the V3 - V4 hypervariable region (Klindworth, et al., 2013). The 25 μL of PCR mixture contained 12.5 μL 2x MyTaq^TM^ Mix (Bioline, UK), ca. 15 ng of template DNA, 1 μL 1:20-diluted UltraPure BSA (50 mg/mL; Invitrogen, USA), 0.4 pM of each primer, and PCR-grade H_2_O to a final volume of 25 µL. The PCR mixture was initially denatured for 3 min at 95 °C, followed by a total of 25 cycles (with each including 30 s at 95 °C, 30 s at 55 °C, and 30 s at 72 °C), with a final extension step at 72 °C for 5 min. Each sample was amplified in two replicates. The 16S PCR products were verified via gel electrophoresis (1.5% agarose) and purified with AmPURE XP beads (Thermofisher, USA) and verified with gel electrophoresis again. Prior to sequencing on Illumina’s MiSeq platformusing V3 600 cycle chemistry (paired-end, 2x 300 bp), library preparation was performed according to manufacturer’s instructions (Illumina, 2013) using My Taq HS Mix 2x (Bioline, UK) and the NexteraXT kit for indexing; the product was subsequently purified by Ampure XP beads and quantified with the Qubit BR (Broad Range) Assay Kit (Thermo Fisher Scientific, USA). The purified PCR products were each diluted to 4 nM before pooling before sequencing. 16S rRNA gene sequences were deposited at the European Nucleotide Archive (ENA) under the accession numbers PRJEB53798 and ERA15908126.

QIIME2 (version 2019.1) (Bolyen, et al., 2019) with cutadapt (version 2.10)(Martin, 2011), DADA2 (1.6.0) (Callahan, et al., 2016), fastQC (0.11.9, <http://www.bioinformatics.babraham.ac.uk/projects/fastqc>), and SILVA release 132 (Quast, et al., 2013) were used for primer removal, removal of untrimmed reads and reads < 50 bp, quality control, quality-filtering, quality-trimming, learning of the error rates, dereplicating, merging of paired end reads, and chimera removal, and taxonomic assignment of raw and de-multiplexed sequence reads.

The barplot and heatmap were created with RStudio version 2022.2.1.461 (RStudio Team, 2020, <http://www.rstudio.com/>). The data frames were converted into single column formats with the *melt()* function (*reshape2* package, Wickham, 2007). A colour assignment was done with the function *brewer.pal()* and the variable *brewer.pal.info()* (*RColorBrewer* package, <https://CRAN.R-project.org/package=RColorBrewer>) before plotting using the function *ggplot()* (*ggplot2(Wickham, 2007)* and *scales* (version 1.1.1. <https://CRAN.R-project.org/package=scales>) packages).

In total, 1,112,572 reads were gathered from all samples derived from both BES reactors under all running mode. Reads per sample ranged from 47,812 to 103,633.

**SI-3 Calculations**

All performance parameters for microbial electrochemical sulfate reduction were calculated for steady state conditions, which was defined when the standard deviation of sulfate removal efficiency of three consecutive sampling points was less than 5%.

Sulfate removal efficiency (*E*_sulfate_) between two sampling points was calculated according to equation S1.

| $E_{\mathrm{sulfate}}=\frac{(C_{SO42-, eff1}+C_{SO42-, eff2})}{2\times C_{SO42-,in}}$ | (Eq.S1) |
| --- | --- |

*C*_SO42−,eff1_ and *C*_SO42−,eff2_ are the sulfate concentrations in the effluent of two consecutive sampling points, *C*_SO42−,in_ is the sulfate concentration in the influent.

Sulfate removal rate (*R*_sulfate_) was calculated according to equation S2.

| $R_{\mathrm{sulfate}}=\frac{v\times\Delta t\times C_{SO42-, in}+C_{SO42-, eff1}\times V-C_{SO42-, eff2}\times\left( V+v\times\Delta t \right)}{V\times\Delta t}$ | (Eq. S2) |
| --- | --- |

*v* is flow rate, *Δt* is the sampling interval of two sampling points, and *V* is the working volume of the BES.

Sulfide formation rate (*F*_sulfide_) was calculated according to equation S3.

| $F_{\mathrm{sulfide}}=\frac{V\times\left( C_{HS-,eff2}- C_{HS-,eff1} \right)+\frac{1}{2}\times\left( C_{HS-,eff2} +C_{HS-,eff1} \right)\times v\times\Delta t}{V\times\Delta t}$ | (Eq. S3) |
| --- | --- |

*C*_HS−,eff2_ is the sulfide concentration in the effluent at the end sampling interval, *C*_HS−,eff1_  is the sulfide concentration in the effluent at the beginning of the sampling interval.

The power consumption was normalized to kilogram reduced sulfate (kWh kg_sulfate_^−1^) according to equation S4.

| $P=\frac{U_{\mathrm{Cell}} \times I}{R_{\mathrm{sulfate}}\times V\times M_{sulfate}}$ | (Eq. S4) |
| --- | --- |

*U*_Cell_ is cell voltage, *I* is current, and $M_{sulfate}$ is the molar mass of sulfate (96.06 g mol^-1^).

The coulombic efficiency (*CE*) was calculated by dividing the charge that accounts for all sulfate being reduced to sulfide with the transferred charge in the same time period (equation S5).

| $CE=\frac{R_{\mathrm{sulfate}}\times\mathrm{HRT}\times V\times z\times F}{\int_{0}^{t} Idt}\times100\%$ | (Eq. S5) |
| --- | --- |

HRT is hydraulic retention time, *V* is the working volume of reactors (0.25 L), *z* is the number of electrons for reducing sulfate to sulfide (8 electrons), *F* is Faradic constant (96485.3 C mol^-1^) *I* is current, *t* is time (being equal to the respectively used HRT).

**SI-4 Comparison with other technologies for sulfate removal**

Kinnunen et al. (2017) treated mine tailings pond water with an average sulfate concentration of 8 g_sulfate_ L^−1^. Application of gypsum precipitation yielded an effluent with 1.4 g_sulfate_ L^−1^ and 2.2 g_sulfate_ L^−1^. Application of ettringite precipitation yielded an effluent with 0.7 g_sulfate_ L^−1^ and 1.8 g_sulfate_ L^−1^. In a corresponding model approach, a pond water feed of 200 m^3^ h^−1^ and power requirements of 300 kW and 450 kW for gypsum and ettringite precipitation were assumed, respectively. The energy consumption was calculated as exemplified in equation S6.

| $P=\frac{300\mathrm{kW}}{(8.0\frac{g_{\mathrm{sulfate}}}{L}-1.4\frac{g_{\mathrm{sulfate}}}{L})\times200\frac{m^{3}}{h}}=0.23\frac{\mathrm{kWh}}{kg_{\mathrm{sulfate}}}$ | (Eq. S6) |
| --- | --- |

Annamalai et al. (2015) treated diluted sulfate-contaminated farming soil with a concentration of 0.5 kg_sulfate_ m^−3^ (2.5 g sulfate per kg soil, 5 g soil resuspended in 25 mL water) with electrokinetic remediation with an *E*_sulfate_ of 96% and an energy consumption of 119 kWh m^−3^. The energy consumption was normalized to kilogram sulfate via equation S7.

| $P=\frac{119 \frac{\mathrm{kWh}}{m^{3}}}{0.5 \frac{\mathrm{kg}_{\mathrm{sulfate}}}{m^{3}}\times0.96}=248.9\frac{\mathrm{kWh}}{\mathrm{kg}_{\mathrm{sulfate}}}$ | (Eq. S7) |
| --- | --- |
|  |  |

Dai et al. (2022) studied bioelectrochemical sulfate reduction in bioelectrochemical systems operated in batch mode. The energy consumption was normalized to kilogram sulfate via equation S8. All input values represent means, which were averaged over the whole batch cultivation.

| $P=\frac{P}{R_{\mathrm{sulfate}}\times V}=\frac{0.0123 kWh}{3.3 \frac{g}{L}\times0.25 L}=14.9\frac{\mathrm{kWh}}{\mathrm{kg}_{\mathrm{sulfate}}}$ |  | (Eq. S8) |
| --- | --- | --- |

**SI-5 Supplementary figures**


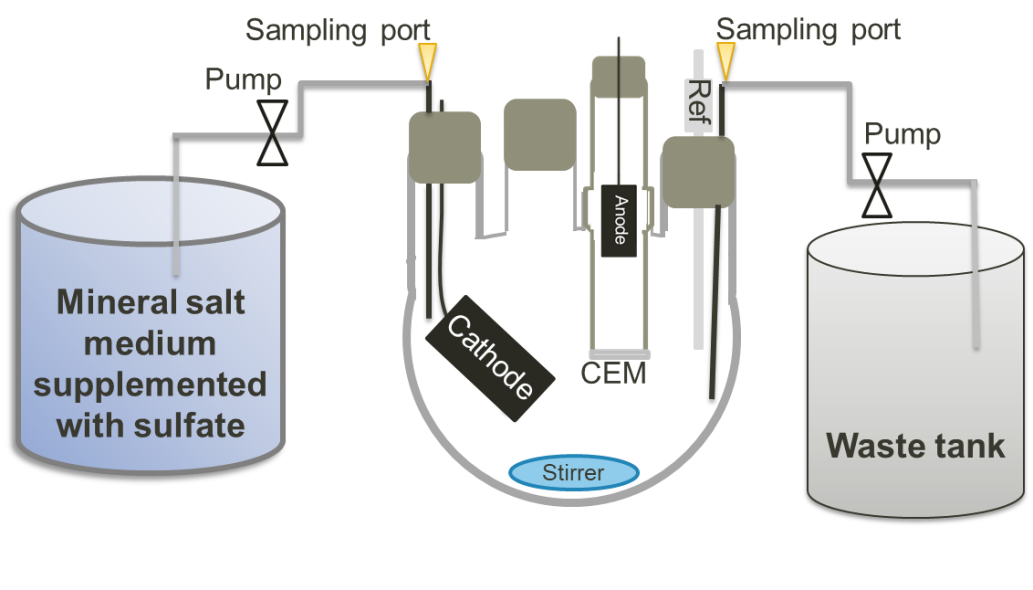


**Figure** **S1** Illustration of the bioelectrochemical system operated in flow mode. Ref: reference electrode, CEM: cation exchange membrane.

**Figure S2** Current densities (*j*) of bioelectrochemical systems operated at different hydraulic retention times (HRT) (a) and different temperatures (*T*) (b). The data was calculated from two reactors and the error bars represent standard deviations (SD) calculated from three consecutive measurements representing steady-state conditions (SD of sulfate removal efficiency <5 %).

**Figure S3** Power consumption (kWh kg_sulfate_^−1^) of bioelectrochemical systems operated at different hydraulic retention times (HRT) (a) and different temperatures (*T*) (b). Data shown are mean values calculated from data of two reactors in steady-state (standard deviation of sulfate removal efficiency < 5%).

**Figure S4** pH and *OD*_600_ of bioelectrochemical systems operated at different hydraulic retention times (HRT) (a) and different temperatures (*T*) (b). The error bars represent standard deviations (SD) calculated from three consecutive measurements representing steady steady-state conditions (SD of sulfate removal efficiency < 5 %).

**Figure S5** Relative abundances of sulfate reducing prokaryotes (SRP), homoacetogens, and microaerobes in liquid phase during application of different hydraulic retention times (HRT) (a) and different temperatures (*T*) (b).

**References**

Annamalai, S., Selvaraj, S., Selvaraj, H., Santhanam, M., and Pazos, M. (2015) Electrokinetic remediation: challenging and optimization of electrolyte for sulfate removal in textile effluent-contaminated farming soil, *RSC Adv* **5**: 81052-81058.

Bin Hudari, M.S., Vogt, C., and Richnow, H.H. (2020) Effect of Temperature on Acetate Mineralization Kinetics and Microbial Community Composition in a Hydrocarbon-Affected Microbial Community During a Shift From Oxic to Sulfidogenic Conditions, *Front Microbiol* **11**: 606565.

Bolyen, E., Rideout, J.R., Dillon, M.R., Bokulich, N.A., Abnet, C.C., Al-Ghalith, G.A., et al. (2019) Reproducible, interactive, scalable and extensible microbiome data science using QIIME 2, *Nat Biotechnol* **37**: 852-857.

Callahan, B.J., McMurdie, P.J., Rosen, M.J., Han, A.W., Johnson, A.J.A., and Holmes, S.P. (2016) DADA2: High-resolution sample inference from Illumina amplicon data, *Nat Methods* **13**: 581-583.

Dai, S., Korth, B., Schwab, L., Aulenta, F., Vogt, C., and Harnisch, F. (2022) Deciphering the fate of sulfate in one- and two-chamber bioelectrochemical systems, *Electrochim Acta* **408**.

Illumina (2013) 16S Metagenomic Sequencing Library Preparation.

Kinnunen, P., Kyllonen, H., Kaartinen, T., Makinen, J., Heikkinen, J., and Miettinen, V. (2017) Sulphate removal from mine water with chemical, biological and membrane technologies, *Water Sci Technol* **2017**: 194-205.

Klindworth, A., Pruesse, E., Schweer, T., Peplies, J., Quast, C., Horn, M., and Glockner, F.O. (2013) Evaluation of general 16S ribosomal RNA gene PCR primers for classical and next-generation sequencing-based diversity studies, *Nucleic Acids Res* **41**: e1.

Martin, M. (2011) Cutadapt removes adapter sequences from high-throughput sequencing reads, *EMBnet.journal* **17**.

McMurdie, P.J., and Holmes, S. (2013) phyloseq: An R Package for Reproducible Interactive Analysis and Graphics of Microbiome Census Data, *PLOS ONE* **8**: e61217.

Quast, C., Pruesse, E., Yilmaz, P., Gerken, J., Schweer, T., Yarza, P., et al. (2013) The SILVA ribosomal RNA gene database project: improved data processing and web-based tools, *Nucleic Acids Res* **41**: D590-596.

Wickham, H. (2007) Reshaping data with the reshape package, *J Stat Softw* **21**: 1-20.
